# The Early ANTP Gene Repertoire: Insights from the Placozoan Genome

**DOI:** 10.1371/journal.pone.0002457

**Published:** 2008-08-21

**Authors:** Bernd Schierwater, Kai Kamm, Mansi Srivastava, Daniel Rokhsar, Rafael D. Rosengarten, Stephen L. Dellaporta

**Affiliations:** 1 Division of Ecology and Evolution, Stiftung Tieraerztliche Hochschule Hannover, Hannover, Germany; 2 Department of Molecular, Cellular and Developmental Biology, Yale University, New Haven, Connecticut, United States of America; 3 Department of Energy, Joint Genome Institute, Walnut Creek, California, United States of America; 4 Center for Integrative Genomics and Department of Molecular and Cell Biology, University of California, Berkeley, California, United States of America; Utrecht University, Netherlands

## Abstract

The evolution of ANTP genes in the Metazoa has been the subject of conflicting hypotheses derived from full or partial gene sequences and genomic organization in higher animals. Whole genome sequences have recently filled in some crucial gaps for the basal metazoan phyla Cnidaria and Porifera. Here we analyze the complete genome of *Trichoplax adhaerens*, representing the basal metazoan phylum Placozoa, for its set of ANTP class genes. The *Trichoplax* genome encodes representatives of Hox/ParaHox-like, NKL, and extended Hox genes. This repertoire possibly mirrors the condition of a hypothetical cnidarian-bilaterian ancestor. The evolution of the cnidarian and bilaterian ANTP gene repertoires can be deduced by a limited number of cis-duplications of NKL and “extended Hox” genes and the presence of a single ancestral “ProtoHox” gene.

## Introduction

Transcription factors of the homeodomain family fulfill important roles during development, cell differentiation and cell proliferation in animals, plants, and fungi [1], [2]. They are characterized by the presence of a conserved DNA binding motif - the 60 amino acids of the homeodomain encoded by the 180 base pairs of the homeobox [3]. In the Metazoa they can be mainly divided into the (super)classes ANTP, PRD, POU, LIM, SIX and TALE, most of which seem to be restricted to the animal kingdom [1], [2].

By far the highest gene diversity can be observed in the ANTP superclass which seems to have been a key factor for the evolution of metazoan bauplans (reviewed in [4]), fueling the radiation of metazoan phyla and regulating the development of body axes, symmetry, and the nervous system. The ANTP superclass consists of the Hox/ParaHox, extended Hox, and NKL genes (their relatedness complies with the above order). All Bilateria possess Hox/ParaHox, NKL, and extended Hox genes [4], [5] and many of them serve conserved functions: The best known examples are the clustered Hox genes which provide positional information along the anterior-posterior body axis during development in the same manner across Bilateria [5]. The conserved role of other ANTP genes is less well understood, due to the lack of comparative functional data. Nevertheless, for example, orthologs of the NKL gene NK2.5 play a conserved role during cardiac development in insects and mammals [2], [4]; and the extended Hox gene Emx is a conserved anterior marker in Bilateria and involved in the development of the anterior parts of the central nervous system [6].

Sequence and linkage data in extant bilaterians indicate that the expansion of the ANTP superclass has taken place by numerous cis-duplications of a single genomic region [4], [7]–[10]. In this scenario an ur-ANTP homeobox gene would have duplicated into the predecessors of Hox/ParaHox, extended Hox and NKL genes. Recent data from cnidarian ANTP gene repertoires and remnants of linkage conform to this scenario of cis-duplications [11]–[15]. Cnidaria possess almost complete repertoires of NKL and extended Hox gene families, but may have branched before a complete Hox system with true anterior, medial, and posterior determinants had evolved [11]–[15]. Recent analyses of a poriferan genome, however, suggest that the origin of the ANTP superclass rather lies in true NKL genes because the sponge *Amphimedon* lacks any Hox/ParaHox-like or extended Hox genes but possesses several clustered NKL genes [16].

The genome of *Trichoplax adhaerens*, representing the early-branching metazoan phylum Placozoa, can give valuable insights into the origin of the ANTP superclass even though its specific phylogenetic position is still debated: While analyses of the mitochondrial genome place the phylum Placozoa at the root of Metazoa [17], genes from the nuclear genome support the divergence of Placozoa from other animals after the divergence of sponges but before (or close to) the cnidarian-bilaterian split [18]. A previous analysis of the ANTP gene content in *Trichoplax* revealed a surprisingly low diversity [19]. With the draft genome of *Trichoplax* in hand [18] we are now able to investigate its ANTP and homeobox gene repertoire in detail. Our analysis of the placozoan genome revealed a greater diversity of ANTP class genes than found in sponges, but less gene complexity than found in other metazoans. The data are consistent with either gene loss in the Porifera from an ancestral metazoan state resembling *Trichoplax*; or a progression from the more primitive state of the demosponge to the more complex eumetazoans, with *Trichoplax* representing a primitive eumetazoan branch. These findings have strong implications for understanding the genomic complexity of the earliest animals and the genetic mechanisms underlying the radiation of metazoan body plans.

## Results and Discussion

Seven homeobox classes comprised of 37 homeobox genes (Table 1, Table S1), including 14 from the ANTP superclass, were identified in the genome of *Trichoplax adhaerens* [18]. Phylogenetic analyses were conducted to infer relationships of these 14 genes to known ANTP families. Bayesian Inference and Maximum Likelihood analyses identify robust grouping for 11 of the 14 genes, representing Hox/ParaHox-like (Gsx), extended Hox (Not, Mnx), and NKL genes (Dlx, 3× NK2, NK5, NK6, Hex and Dbx/Hlx) (Figures S1 & S2). Jakob *et al.*, 2004 [20] suggested that the Hox/ParaHox-like gene, *Trox-2* (Gsx), may represent an ancestral ProtoHox gene, which is consistent with our extended analysis identifying *Trox-2* as the only Hox/ParaHox type gene in *Trichoplax*. The remaining three ANTP genes (Hox/ext.Hox-related, NK-related1 & 2) are highly derived and may be the result of recent duplication and rapid divergence (Table 1, Figure S1 & S2). Overall, the ANTP repertoire of *Trichoplax adhaerens* is more complex than previously thought [19]. These data are consistent with a position of Placozoa ancestral to the cnidarian-bilaterian lineage.

**Table 1:** The homeobox gene complement of *Trichoplax adhaerens* shows a slightly higher diversity of the homeobox classes ANTP, PRD and LIM.

	*Trichoplax*	*Nematostella*	*Amphimedon*
**ANTP**	14	78	8
NKL	NK2, NK5, NK6, Hex, Dlx, Dbx/Hlx+2 NK-related genes	all (bilaterian) families except Tlx	NK2(2/3), NK6, Msx, BarH-related, Hex-related, Tlx-related
Ext. Hox	Not, Mnx	all families except Eng and possibly Vax	-
Hox/ParaHox	Gsx+1 gene with some affinity to ext. Hox and Hox/ParaHox	Gsx, anterior Hox, posterior Hox/Cdx-like+several genes with unclear relation to bilaterian Hox/ParaHox genes	-
**PRD**	9 (Arx, Ebx/Arx-like, Pax3-like, PaxB, Prd/Pax-like, Pitx, Gsc, Otp)	33 (PaxA, B, C, Pax6-like, Arx, Rx, Pitx, Otx, Otp, Gsc & several unassigned)	9 (Arx, PaxB, Rx, OG12)
**POU**	2 (Pou3, 4,)	5 (POU1, 3, 4, 6)	4 (POU 1, 6, 2–5)
**LIM-HD**	5 (LIM1, 1/5, 3/4, 2/9, Isl)	6 (Lim1, 2, 3/4 Awh, Isl)	3 (LIM3, Lin-11, Isl)
**SIX**	2 (Six1/2, Six3/6)	5 (Six2, 3, 4, 4/5)	1 (Six1/2)
**TALE**	4 (PBX/PBC, Pknox, Irx, Meis)	7 (PBC, TGIF, Meis, Irx)	6 (Meis, PBC, Irx)
**HNF**	1	1	-
**Total**	37	134	31
			
**Ref.**	This study	[13]	[16]

Orthology assignment of the *Trichoplax* homeobox genes is based upon BLAST hits in the case of non-ANTP genes. ANTP genes have been classified according to phylogenetic analyses shown in Figure S1 & S2.

**Figure 1:**
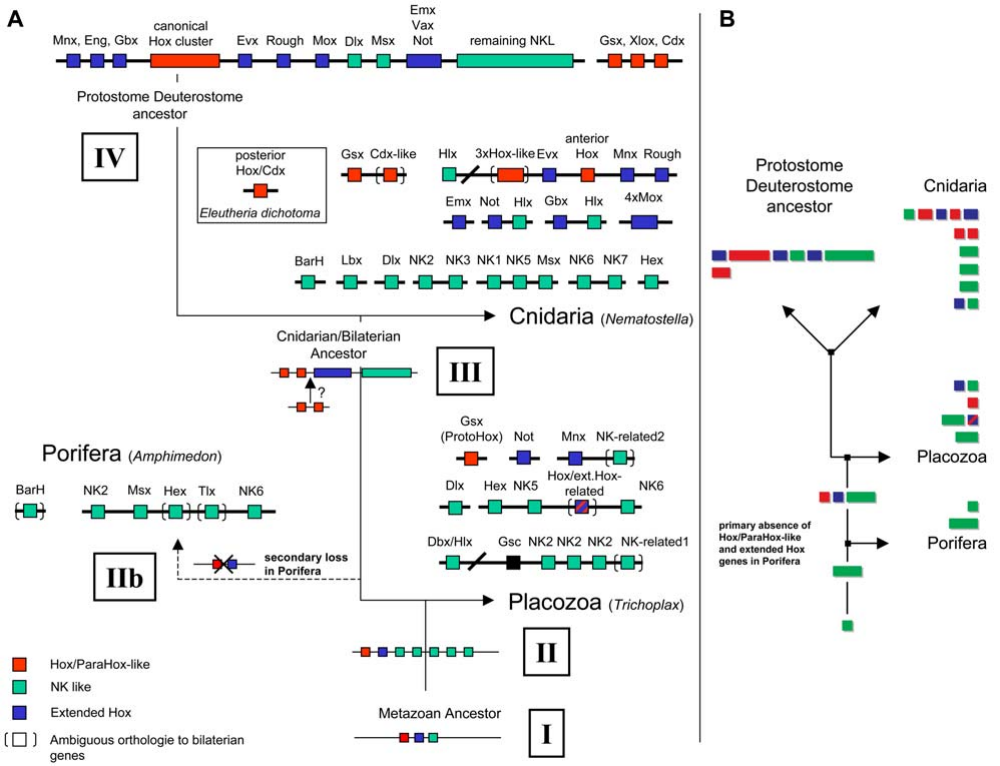
Two possible scenarios for the evolution of the ANTP superclass in Metazoa. A: The Placozoa basal scenario. I The metazoan ancestor may have already possessed clustered single representatives of NKL, extended Hox and Hox/ParaHox-like genes. II Expansion of the NKL subclass. This situation is exemplified by the ANTP gene complement in *Trichoplax adhaerens*, which harbors several clustered NKL genes, two genes of the extended Hox and a single Hox/ParaHox-like gene which is a true Gsx ortholog and may be the founder of the Hox/ParaHox-like subclass of ANTP genes. IIb Based on the assumption that Placozoa are basal within extant metazoan phyla, Porifera must have lost extended Hox, Hox/ParaHox-like and some NKL genes. An alternative scenario that Porifera are most basal would in turn require loss of some NKL genes in Placozoa (e.g. Msx and Bar/Bsh related genes). III Further expansion of ANTP genes in a cnidarian-bilaterian ancestor leading to almost complete repertoires of NKL and extended Hox genes. Occurrence of anterior Hox and posterior Hox/Cdx-like genes. The assumption that a Gsx type gene was the founder of Hox/ParaHox-like genes requires that Gsx and a posterior Hox/Cdx-like gene were part of an ancestral ANTP mega-array for which we still lack any evidence in extant species (question mark in III). In urochordates, however, we find one example of a ParaHox gene (Cdx) which is tightly linked to a Hox gene [23]. IV occurrence of a true canonical Hox System and complete ParaHox cluster in Bilateria. Linkages other than for *Trichoplax adhaerens* are based upon[11], [13], [15], [16]. In the case of *Nematostella* some linkages have been deduced from the current JGI genome assembly. The proposed ANTP mega-array in a cnidarian-bilaterian ancestor has been deduced from [7], [8], [10], [12], [15]. Remark: For completeness the black square in the *Trichoplax* genome represents the PRD class gene Gsc which is tightly linked to NKL genes. B: A Porifera basal scenario (simplified) for the Evolution of ANTP genes leading to crown group diploblasts and a putative protostome deuterostome ancestor: starts with a single (proto)NK gene and an initial expansion of the NKL subclass. Hox/ParaHox-like and extended Hox genes are an invention of the lineage leading to Placozoa, Cnidaria and Bilateria.

Comparing the homeobox gene composition of *Trichoplax* to genomes of the poriferan *Amphimedon* and the cnidarian *Nematostella* reveals that the Placozoa possess significantly less homeobox diversity than Cnidaria but a similar diversity as found in Porifera (Table 1). While no significant differences in diversity are found for PRD, POU, LIM, SIX, and TALE class genes, the most striking difference is the absence of any extended Hox or any Hox/ParaHox-like genes in the Porifera (as represented by *Amphimedon* [16]). Furthermore *Amphimedon* has the smallest ANTP-class gene repertoire with 8 (all NK related) genes [16], while *Trichoplax* harbors 14 and *Nematostella* close to 80 ANTP class genes [13]. Nearly all ANTP genes in *Trichoplax adhaerens* show a clear relationship to known homologs from bilaterians (Figure S1 & S2) and are organized in two tight NK clusters, with the exception of the unclustered extended Hox genes and the Gsx type gene, *Trox-2* (Figure 1). Comparison of the linkage patterns in sponges [16], cnidarians [11], [13], [15] and bilaterians [4], [7], [8], [10] suggests that most or all ANTP gene families have emerged from a single ancestral region and the remnants of linkage in *Trichoplax* conform to this scenario (Figure 1). Moreover, many of the remaining homeobox classes (Table 1) are reciprocally linked in the *Trichoplax* genome (Figure S3, Table S1), suggesting also ancient linkage of the predecessors of all metazoan homeobox classes (cf.[21], [22]).

An enigma remains: In all investigated extant species, including *Trichoplax* (Figure 1), the ParaHox gene Gsx is never linked to Hox, extended Hox or NKL genes (e.g. [4], [5], [13]), suggesting that Gsx was never part of the ANTP array, but rather originated through trans-duplication of a predecessor of the Hox cluster, together with one or two of the remaining ParaHox cluster genes (cf. [4], [5]). However, if a Gsx type gene represents the ancestral ProtoHox it must have been part of this array since Hox genes originally were part of it [7], [8], [10]–[13], [15]. Nevertheless, in the urochordate *Oikopleura* we find linkage between the ParaHox gene Cdx and the Hox gene Hox1 [23] which is to be expected if distinct Hox and ParaHox genes resulted from cis-duplication and were both part of the ANTP array before the ParaHox genes were translocated in most species. Together with the fact that the Gsx type gene Trox-2 is the only Hox/ParaHox gene in *Trichoplax* we may speculate that it indeed represents the ProtoHox gene which founded the Hox/ParaHox genes, although we cannot completely exclude the possibility that *Trichoplax* has lost other Hox/ParaHox genes. Unfortunately gene trees may not be able to resolve this problem because relationships (clades) beyond the family level of ANTP genes (e.g. the Gsx, NK6, Evx etc. family) are, in most cases, weakly supported (e.g. Figure S1 & S2; cf. [4], [5]). In other words, there is no real confidence which Hox/ParaHox clade is basal to the others.

Understanding the evolution of ANTP genes requires interpreting the data in a phylogenetic context. Unfortunately, the relationships between the four extant lower metazoan phyla, Placozoa, Porifera, Cnidaria, and Ctenophora, remain contentious. Total evidence analyses as well as mitochondrial genome data suggest a basal branch for Placozoa relative to all other diploblast phyla [17], [24]–[26], while extensive nuclear gene phylogenies [18] place Placozoa as the sister taxon to the Cnidaria and Bilateria. Depending on the placement of Placozoa, two scenarios for ANTP gene evolution come to mind: (1) Porifera branching first would support the hypothesis of an Urmetazoan harboring a set of NKL genes only, which later in eumetazoans (all non-poriferan metazoans) diverged into the different ANTP gene families (cf.[7], [10]) (Figure 1). (2) Placozoa branching first among extant phyla supports a scenario in which the ancestral Urmetazoan harbored a set of NKL, Hox/ParaHox-like and extended Hox genes (Figure 1) (cf.[7], [10]). In this view Porifera have lost Hox/ParaHox related and extended Hox genes. This is congruent with a recent hypothesis based on the alternative interpretation of gene trees [27]. Both scenarios as well as all recent phylogenetic analyses, are congruent with the hypothesis that Placozoa mirrors the closest condition for an ancestral eumetazoan genome from which the situation in Cnidaria and Bilateria, can be derived [18].

While the final answer to the origin of Metazoa, the basal metazoan bauplan and (homeobox) gene content, might never be found [28], overall evidence is congruent with the view that an ancestral metazoan ANTP array harbored at least one ortholog each of Hox/ParaHox-like, NK, and extended Hox genes. During anagenetic evolution into crown-group placozoans, cnidarians and bilaterians, gene duplications within each ANTP family then gave rise to a wide array of NKL genes in all extant metazoans, most extended Hox genes in the cnidarian-bilaterian lineage, Hox-like genes in the Cnidaria, and true Hox and ParaHox gene clusters in Bilateria (Figure 1). Independent of the above models, our data support the view that ANTP gene evolution was driven by three major events: (A) an initial expansion of the NKL subclass, (B) the expansion of the extended Hox, and (C) a Gsx type gene giving rise to all Hox/ParaHox and related genes.

## Materials and Methods

### Genome sequencing

The so-called “Grell” strain has been in culture in Hannover as clonal lineage since 1999 [25], and at Yale University since 2001. From this clone, which is the same used in earlier reports on placozoan genetics [17], [19], [25], [29], genomic DNA was prepared for genomic library construction at JGI. For details on shotgun sequencing and bioinformatic analyses see [18].

### Identification of Trichoplax homeodomain genes

A database of the 8× coverage of assembled scaffolds of the *Trichoplax adhaerens* genome was queried locally with a list of 1,063 known homeodomain protein motifs (available upon request), using the translated blast algorithm (tblastn) filtered at a low-stringency e-value of 0.001. At this stage of the analysis, some false positive hits were accepted in favor of detecting all true positives. These false hits would later be sifted out by a second reciprocal blast search as described below. The tblastn hits were sorted by genomic coordinates yielding 403 unique HSPs (blast High Scoring Pair) representing putative Trichoplax homeodomain homologs.

For the reverse blast analysis, four kilobases of genomic DNA sequence encompassing each of the tblastn HSPs was retrieved from the *Trichoplax* genome database using custom perl scripts (available upon request). These 4 kb subcontigs were individually queried against a local swissprot database using the translated blastx program, filtered at a more stringent e-value of 10∧-5. The blastx output for each contig was parsed using custom scripts along with the identity of its top blast hit and e-value (available upon request). Forty-seven loci in the *T. adhaerens* genome were confirmed to have strong blast similarity to known homeodomain containing proteins, of which 37 were confirmed to harbor a homeobox. Gene linkage and clustering were identified based on scaffold coordinates.

### Phylogenetic Analysis

Blast searches identified 14 of the 37 loci as ANTP class homeobox genes. The remaining 23 putative homeobox genes were roughly classified based on Blast results. The 14 putative ANTP class genes were further analyzed to infer clear relationships to known ANTP gene families. In the cases where the homeodomain is encoded by more than one exon, the homeodomain amino acid sequences were predicted by Genscan and GenomeScan [30], [31].

The 60 amino acids of the homeodomain were aligned with ClustalW implemented in MEGA 3.1 [32]. Bayesian Inference implemented by Mr.Bayes v3.1.2 [33], and maximum-likelihood analyses were conducted to infer phylogenetic relationships to known ANTP families. Mr.Bayes employed the JTT model of amino acid substitution assuming the presence of invariant sites and a gamma distribution approximated by four different rate categories to model rate variation between sites. MrBayes was run with four chains for 5 million generations sampling every 1000 generations. The likelihoods of the generations were examined to estimate the beginning of stationary phase and trees after the first 1.25 million generations were used to create a consensus. Maximum Likelihood analyses were performed using PHYML v.2.4.4 [34] with the JTT model and a gamma distribution approximated by four rate categories. Tree robustness was assessed by 1000 bootstrap resamplings. The resulting trees of Bayesian and Maximum Likelihood analyses were rooted on midpoint. The dataset comprised the 14 putative ANTP genes from *Trichoplax* together with representatives of known ANTP families (cf. [2], [4], [7], [10], [12], [14], [16] of Bilateria, Cnidaria and Porifera from public databases (166 homeodomains in total).

*Nematostella* sequences were taken from a published dataset [11], [12] except for NvBarx (ABG67788), NvHD065 (ABC16641) and NvHlxB (ABG67796). *Amphimedon* sequences were taken from [16]. Accession numbers for the remaining sequences are available as supplemental data (Text S1). *Trichoplax* sequences have been deposited to GenBank (EU700377-EU700390 for the ANTP genes; EU700377-EU700413 for all homeobox genes) and are also available as supplemental data (Table S1), together with genomic scaffold coordinates.

## Supporting Information

Figure S1 Bayesian analysis reveals robust grouping for 11 of 14 placozoan ANTP genes to known ANTP genes from Cnidaria, Porifera, and Bilateria. Genes included in the analysis represent Placozoa (*Trichoplax adhaerens* Ta), Cnidaria (*Nematostella vectensis* Nv, *Eleutheria dichotoma* Ed, *Acropora Formosa* Af), Porifera (*Amphimedon queenslandica* Aq), protostomes (*Drosophila melanogaster* Dm, *Anopheles gambiae* Ag, *Discocelis tigrina* Dt, *Schistocerca gregaria* Sg), and deuterostomes (*Branchiostoma floridae* Bf, *Danio rerio* Dr, *Homo sapiens* Hs, *Oikopleura dioica* Od, *Strongylocentrotus purpuratus* Sp, *Xenopus laevis* Xl). Genes of the NKL subclass are framed in yellow, extended Hox genes in blue and Hox/ParaHox-like genes in red. *Amphimedon*, *Nematostella* and *Trichoplax* genes are color coded. Tree rooted on midpoint.
(0.09 MB PDF)

Figure S2 Maximum Likelihood analysis with the same dataset as above. Tree rooted on midpoint.
(0.07 MB PDF)

Figure S3 Linkage of non-ANTP homeobox genes in *Trichoplax adhaerens*. In the current assembly of the Trichoplax genome it is evident that the genes of the homeobox classes ANTP, PRD, LIM, TALE and Hnf are reciprocally linked. This supports the view that at least the founders of most metazoan homeobox classes have been ancestrally clustered (cf.[21], [22]). Only distances between two genes in the megabase range are given. All other distances are ≤300 kb.
(0.05 MB PDF)

Table S1 Sequences of the *Trichoplax* homeodomains/homeoboxes as well as other conserved motifs and genomic scaffold coordinates.
(0.04 MB XLS)

Text S1 Accession numbers of the sequences used for phylogenetic analyses.
(0.06 MB DOC)
