# Supplementary figures and images for: The Early ANTP Gene Repertoire: Insights from the Placozoan Genome
Source: PLoS One. 2008 Aug 21;3(8):e2457. doi: 10.1371/journal.pone.0002457 (PMC2515636; doi:10.1371/journal.pone.0002457)

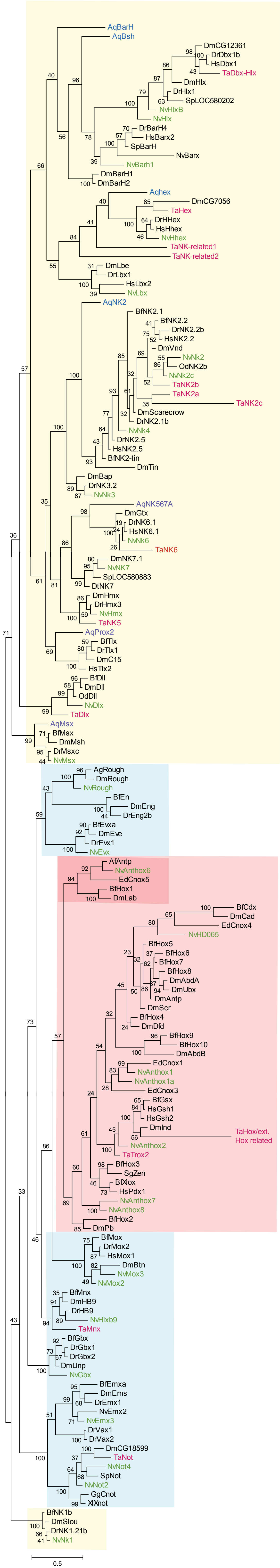

Supplement: Figure S1 — Bayesian analysis reveals robust grouping for 11 of 14 placozoan ANTP genes to known ANTP genes from Cnidaria, Porifera, and Bilateria. Genes included in the analysis represent Placozoa (Trichoplax adhaerens Ta), Cnidaria (Nematostella vectensis Nv, Eleutheria dichotoma Ed, Acropora Formosa Af), Porifera (Amphimedon queenslandica Aq), protostomes (Drosophila melanogaster Dm, Anopheles gambiae Ag, Discocelis tigrina Dt, Schistocerca gregaria Sg), and deuterostomes (Branchiostoma floridae Bf, Danio rerio Dr, Homo sapiens Hs, Oikopleura dioica Od, Strongylocentrotus purpuratus Sp, Xenopus laevis Xl). Genes of the NKL subclass are framed in yellow, extended Hox genes in blue and Hox/ParaHox-like genes in red. Amphimedon, Nematostella and Trichoplax genes are color coded. Tree rooted on midpoint. (0.09 MB PDF) [file pone.0002457.s001.pdf]

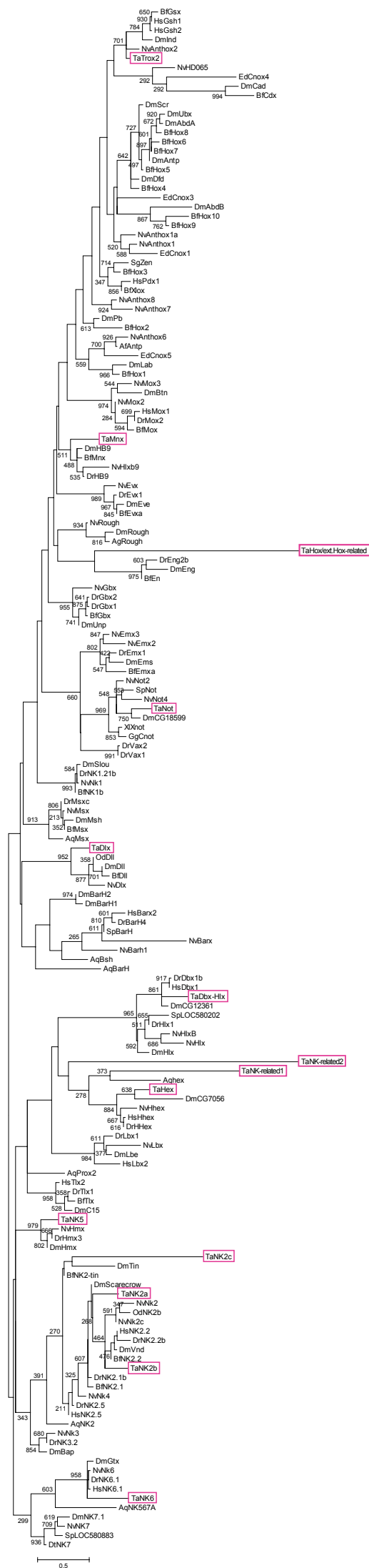

Supplement: Figure S2 — Maximum Likelihood analysis with the same dataset as above. Tree rooted on midpoint. (0.07 MB PDF) [file pone.0002457.s002.pdf]

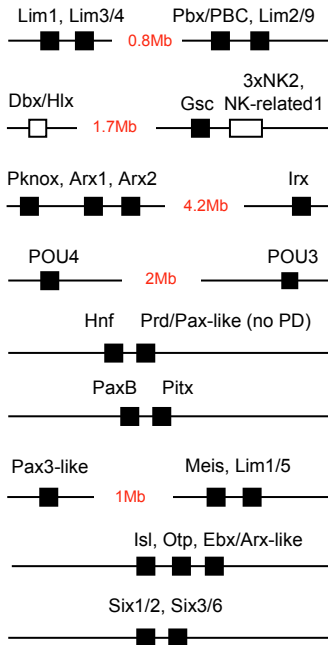

Supplement: Figure S3 — Linkage of non-ANTP homeobox genes in Trichoplax adhaerens. In the current assembly of the Trichoplax genome it is evident that the genes of the homeobox classes ANTP, PRD, LIM, TALE and Hnf are reciprocally linked. This supports the view that at least the founders of most metazoan homeobox classes have been ancestrally clustered (cf.[21], [22]). Only distances between two genes in the megabase range are given. All other distances are ≤300 kb. (0.05 MB PDF) [file pone.0002457.s003.pdf]
