# Supplementary material for: The Early ANTP Gene Repertoire: Insights from the Placozoan Genome
Source: PLoS One. 2008 Aug 21;3(8):e2457. doi: 10.1371/journal.pone.0002457 (PMC2515636; doi:10.1371/journal.pone.0002457)
Supplement: Text S1 — Accession numbers of the sequences used for phylogenetic analyses. (0.06 MB DOC) [file pone.0002457.s005.doc]

| **Name** | **Accession#** | **Alternative Name/Remarks** |
| --- | --- | --- |
| AfAntp | Q7M3U4 |  |
| AgRough | XP_312583 |  |
| BfCdx | AAC39017 |  |
| BfDll | P53772 |  |
| BfEmxA | AAF76327 |  |
| BfEn | AAB40144 | AmphiEn |
| BfEvxa | AAK58953 | amphiEvxA |
| BfEvxb | AAK58954 | amphiEvxB |
| BfGsx | AAC39015 |  |
| BfHox1 | BAA78620 |  |
| BfHox2 | BAA78621 |  |
| BfHox3 | CAA48180 |  |
| BfHox4 | BAA78622 |  |
| BfHox5 | CAA84517 |  |
| BfHox6 | CAA84518 |  |
| BfHox7 | CAA84519 |  |
| BfHox8 | CAA84520 |  |
| BfHox9 | CAA84521 |  |
| BfHox10 | CAA84522 |  |
| BfMnx | AAG33015 |  |
| BfMox | AAM09689 | amphimox |
| BfMsx | CAA10201 |  |
| BfNK1b | CAD83854 | amphiNK1b |
| BfNk2-2 | AAD01958 | Nk2-2 |
| BfNkx2-1 | AAC35350 | Nkx2-1 |
| BfNK2-tin | AAM90855 |  |
| BfTlx | CAD83853 | AmphiTlx |
| BfXlox | AAC39016 |  |
| DmAbdA | NP_476693 |  |
| DmAbdB | CAB57859 |  |
| DmAntp | AAA70214 |  |
| DmBarH2 | AAA28383 |  |
| DmBarH1 | NP_523387 | B-H1 |
| DmBap | AAQ73809 | bagpipe/NK3 |
| DmBtn | CAA10727 | buttonless |
| DmCad | NP_476954 |  |
| DmC15 | AAY51522 |  |
| DmCG12361 | NP_647677 | Dbx |
| DmCG18599 | AAF55524 | Not |
| DmCG7056 | AAF55844 | Hex |
| DmDll | AAB24059 |  |
| DmEms | CAA35965 | empty spiracles |
| DmEn | AAA65478 | engrailed |
| DmEve | CAA28784 | even-skipped |
| DmHB9 | AAF50503 | exex |
| DmGtx | AAF33780 | Gtx/NK6 |
| DmHlx | P10035 | H2.0 |
| DmHmx | AAF55433 |  |
| DmInd | NP_996087 |  |
| DmLab | NP_476613 |  |
| DmLbe | CAA70056 | ladybird early |
| DmMsh | CAA59680 |  |
| DmNK7.1 | NP_731904 |  |
| DmPb | CAA45272 |  |
| DmRough | A30046 |  |
| DmScarecrow | EAA45970 | scro |
| DmScr | NP_524248 |  |
| DmSlou | P22807 | slouch |
| DmTin | NP_524433 |  |
| DmUbx | NP_996219 |  |
| DmUnp | AAF58968 | unplugged |
| DmVnd | NP_001036253 |  |
| DrBarH4 | NP_001018139 |  |
| DrEmx1 | NP_937787 |  |
| DrEng2b | NP_571115 |  |
| DrEvx1 | NP_571324 |  |
| DrGbx1 | NP_777286 |  |
| DrGbx2 | NP_694496 |  |
| DrHhex | NP_571009 |  |
| DrHlx1 | NP_997866 |  |
| DrHB9 | NP_001009885 |  |
| DrHmx3 | NP_571709 |  |
| DrLbx1 | NP_001007135 |  |
| DrMox2 | XP_684924 |  |
| DrMsxC | CAD60842 |  |
| DrNKx1.21b | NP_998713 |  |
| DrNk2.1b | NP_571851 |  |
| DrNK2.2b | NP_001007783 |  |
| DrNK3.2 | NP_835233 | bapx1 |
| DrNK2.5 | NP_571496 |  |
| DrNK6.1 | NP_001002475 |  |
| DrTlx1 | NP_739571 |  |
| DrVax1 | NP_919391 |  |
| DrVax2 | NP_919390 |  |
| DtNK7 | CAC19387 |  |
| EdCnox-1 | ABE68629 |  |
| EdCnox-3 | ABE68630 |  |
| EdCnox-4 | ABE68631 |  |
| EdCnox-5 | ABE68632 |  |
| GgCnot | NP_990685 | Gnot1 |
| HsBarx2 | NP_003649 |  |
| HsHhex | AAH14336 |  |
| HsLbx2 | NP_001009812 |  |
| HsNK2.2 | NP_002500 |  |
| HsNK2.5 | NP_004378 |  |
| HsNK6.1 | NP_006159 |  |
| HsTlx2 | NP_057254 |  |
| HsGsh1 | NP_663632 |  |
| HsGsh2 | NP_573574 |  |
| HsPdx | NP_000200 |  |
| NvAnthox1 | ABG67762 | HoxF |
| NvAnthox1a | ABG67761 | HoxE |
| NvAnthox2 | ABG67765 | Gsx |
| NvAnthox6 | AAD39348 | Hox6, HoxA |
| NvAnthox7 | ABG67758 | HoxC |
| NvAnthox8 | ABG67759 | Anthox8a, HoxDa |
| NvBarh1 | ABG67791 | Bsh-like 3, NvHD097 |
| NvBarx | ABG67788 | NvHD41 |
| NvDlx | ABG67787 |  |
| NvEmx2 | ABG67798 | EmxA |
| NvEmx3 | ABG67799 | EmxB |
| NvEvx | ABG67773 | anth-eve |
| NvGbx | ABG67771 |  |
| NvHD065 | ABG67763 | Xlox/Cdx |
| NvHhex | ABG67786 | Hex |
| NvHlx | ABG67797 | HlxC |
| NvHlxB | ABG67796 |  |
| NvHlxb9 | ABG67770 | Mnx |
| NvHmx | ABG67783 | NK5 |
| NvLbx | ABG67785 |  |
| NvMox2 | ABG67766 | MoxA |
| NvMox3 | ABG67767 | MoxB |
| NvMsx | ABG67792 | MsxA |
| NvNK1 | AAP88429 | NK-1.Nv |
| NvNK2 | AAP88430 | NK-2.Nv |
| NvNk2c | ABG67779 | NK2-VndD |
| NvNK3 | AAP88431 | NK-3.Nv |
| NvNK4 | AAP88432 | NK-4.Nv |
| NvNK6 | ABG67784 |  |
| NvNK7 | ABB86440 |  |
| NvNot2 | ABG67803 | Not-like B |
| NvNot4 | ABG67804 | Not-like C |
| NvRough | ABG67772 |  |
| OdDll | AAT39335 |  |
| OdNK2b | AAW24456 |  |
| SgZen | CAB61208 | Hox3 |
| SpBarH | XP_798061 | BarH like 1 |
| SpNot | NP_999727 |  |
| SpLOC580883 | XP_786006 | NK7 |
| SpLOC580202 | XP_785368 | Hlx |
| XlXnot | CAA79629 |  |
